# Egoism or Altruism? The Influence of Cause-Related Marketing on Customers’ Extra-Role Behavior

**DOI:** 10.3389/fpsyg.2022.799336

**Published:** 2022-03-01

**Authors:** Zhang Hui, Hu Wenan

**Affiliations:** ^1^School of Business Administration, Shandong University of Finance and Economics, Jinan, China; ^2^Shandong Institute of Talent Development Strategy, Shandong University, Jinan, China

**Keywords:** egoistic cause-related marketing, altruistic cause-related marketing, customer extra-role behavior, customer promotion focus, customer prevention focus

## Abstract

Based on attribution theory and regulatory focus theory, this paper discusses the influence mechanism of cause-related marketing on customers’ extra-role behavior and the moderating effects of customer promotion focus and customer prevention focus. The results show that egoistic cause-related marketing (ECRM) has a negative impact on customer extra-role behavior, while altruistic cause-related marketing has a positive impact on customer extra-role behavior. Customer promotion focus has a significant positive moderating effect on the negative impact of ECRM on customer extra-role behavior; customer promotion focus also has a significant positive moderating effect on the positive effect of altruistic cause-related marketing on customer extra-role behavior. There is a significant negative moderating effect of customer prevention focus on the negative impact of ECRM on customer extra-role behavior, while there is a significant positive moderating effect of customer prevention focus on the positive impact of altruistic cause-related marketing on customer extra-role behavior.

## Introduction

With the continuous development of the market economy, the competition among enterprises has become increasingly fierce, and the awareness of customer citizenship has continued to increase, which puts forward higher requirements for enterprises to fulfill their social responsibilities (Lu et al., 2020). Especially in the context of the COVID-19, how to balance enterprise operation and social responsibility has become a difficult problem for entrepreneurs (Shen et al., 2017). Cause-related marketing (CRM) is an effective way for enterprises to break through the marketing dilemma and enhance business value (Guerreiro et al., 2016). It also provides a good opportunity for enterprises to fulfill their social responsibilities (Mo, 2021). Enterprises develop CRM activities to establish a good corporate image in the eyes of customers. For example, Nongfu Springs called for “Buy a bottle of Nongfu Springs, donate 1 cent for the Hope Project,” Ali Group’s “Ant Forest” and WeChat campaign.

In recent years, CRM as a multi-win marketing model that effectively integrates corporate benefits, social welfare and consumer benefits has gradually become a hot topic in the corporate and academic circles. CRM refers to the process in which a company donates a certain percentage of the income from product sales to the charitable organization that it cooperates with to support related public welfare undertakings (Varadarajan and Mcnon, 1988). Therefore, CRM is not only a commercial activity that can generate economic profits, but also a social marketing strategy that can bring social value to the enterprise (Sun and Wang, 2017). However, there are also some customers questioning the fundamental motives of the enterprise’s CRM, believing that when the enterprise conducts CRM activities in name, it is actually a “show,” which ultimately leads to damage to the corporate image (Sun and Wang, 2016). For example, after the “Red Cross Scandal,” customers’ trust and participation in charity activities carried out by the enterprise was significantly reduced. It can be seen that CRM connects corporate social donations and product sales, and its success depends on customers’ motivational perception, attitude and behavior (Jiang and Zheng, 2017). Among them, the customer’s perception of self-interest or altruistic motives of enterprise CRM is a key factor that affects customer attitudes and behaviors (Bi et al., 2016). The higher the donation level of enterprise CRM is, the stronger the attribution of customers to their altruistic motives is, and the easier it is for customers to have the willingness to buy products (Koschate et al., 2012). In addition, related studies have shown that customer behavior is very important to companies. Positive customer behavior is conducive to cost savings and enterprise performance, while negative customer behavior is not conducive to the sustainable growth of the enterprise (Lv and Wei, 2012). Relevant scholars further divide customer behavior into intra-role behavior and extra-role behavior based on the role of “organizational employee” or “part-time employee” played by customers in the process of participating in service production (Groth, 2005). As a spontaneous behavior of the customer, the customer extra-role behavior exceeds the needs of the normal service production and delivery of the enterprise. It is specifically manifested in the behavior of propagating a good image of the enterprise to others or recommending related products and services of the enterprise.

However, the existing research results are based on the relevant theories of social psychology, and they mostly focus on exploring the influence mechanism of enterprise CRM on customer attitudes (Guerreiro et al., 2015; Savary et al., 2015; Hanks et al., 2016; Sung et al., 2020; Zhang et al., 2020; Ye et al., 2021), ignoring the influence mechanism of enterprise CRM on customer behavior, especially enterprise CRM plays an important role in customer extra-role behavior, so it is difficult to clarify the transformation mechanism between customer attitudes and customer behaviors, and promote the success of enterprise CRM. In view of this, this research is based on attribution theory and adjustment focus theory. It mainly focuses on the two issues of whether enterprise CRM can stimulate customers to take extra-role behavior and whether different customer adjustment focus characteristics can affect the influences of enterprise CRM activities. To explore the influence mechanism of enterprise CRM on customer’s extra-role behavior in Chinese context, and the contingent effect of the personal characteristic of customer adjustment focus on the relationship between CRM and customer extra-role behavior.

## Related Research Reviews

### Regulatory Focus Theory

Regulatory focus refers to a specific tendency or way that an individual shows in the process of controlling and changing his or her own thoughts or reactions in order to achieve a certain goal (Avnet and Higgins, 2006), and individual preferences and specific decision-making processes will be affected by their own regulatory focus (Chang et al., 2020). The regulatory focus theory distinguishes two different self-regulation methods of promotion focus (PROF) and prevention focus (PREF) (Higgins, 1998). Promotion focus reflects positive results related to passion, hope and achievement, while prevention focus reflects negative results related to responsibility, safety and obligation (Crowe and Higgins, 1997). Among them, promotion focus individuals have strong adventurous characteristics, pay more attention to information about positive results such as progress and achievements, tend to obtain maximum benefits at all costs, prefer to choose desire-approaching strategies, and thus exhibit positive and enterprising behaviors (Liu, 2015). However, the prevention focus individuals have obvious conservative characteristics who are vulnerable to negative information, and value obligations and responsibilities. They often choose to abandon significant gains to ensure that they are not lost. Therefore, they prefer to use vigilance-avoidance strategies and tend to adopt risk aversion behaviors (Sha et al., 2013). It can be seen that there are differences between the two types of regulatory focus individuals in terms of psychological appeal, goal results and emotional experience. The promotion focus individuals pay attention to the positive effects and experience more emotions related to happiness and depression in the face of the results, while the prevention focus individuals pay attention to negative effects, fulfill personal responsibilities and obligations and experience more emotions related to calm or anxiety in the face of results (Li et al., 2014).

Sha et al. (2013) found that customer regulatory focus had a significant moderating effect on the relationship between commercial agglomeration impressions and customer identification; based on the data of 416 online shopping consumers, Li et al. (2014) found that customer regulatory focus played a moderating role in the positive impact of online service scenarios on customer flow experience; Liu (2015) further examined the different effects of tourists’ promotion and prevention focus on purchase intentions; Chang et al. (2020) explored the influence of customer regulatory focus on hedonic upgrades in the context of consumers buying different products of the same brand; Huang et al. (2020) explored the influence of consumer regulatory focus on their willingness to adopt smart hardware, and found that if promotion focus consumers were promoted to adopt result simulation strategies, while the prevention focus consumers adopted recall analogy strategies, it can effectively improve consumers’ willingness to adopt smart hardware. It can be seen that the existing research results are mostly based on the regulatory focus theory to explore the influence of different regulatory focus characteristics on individual purchase intention or behavior (Sha et al., 2013; Li et al., 2014; Liu, 2015; Chang et al., 2020; Huang et al., 2020), and the relevant applications of regulatory focus in the field of enterprise CRM and customer extra-role behavior are less involved. In view of this, this research is based on the regulatory focus theory to explore the moderating effect of customer promotion focus and prevention focus on the relationship between enterprise CRM and customer extra-role behavior.

### Enterprise Cause-Related Marketing

Varadarajan and Mcnon (1988) proposed the definition of CRM for the first time, believing that CRM is a marketing activity with special meaning. When a product exchange occurs between a company and a consumer, the company will donate a certain percentage or amount income to the relevant charity. It can be seen that CRM is a corporate activity that integrates charity and promotion with the purpose of urging enterprises to obtain more social and economic benefits (Luo and Lv, 2019). In addition, customers can experience the emotion of “changing the world” by purchasing related products or enjoying services in enterprise CRM activities, which can create more value for customers to a certain extent (Porter and Kramer, 2011). When customers perceive that the company’s motivations are not sincere in the CRM activities, they will reduce their reputation and loyalty to the company’s brand (Pharr and Lough, 2012). In summary, CRM is a “double-edged sword.” Companies should fully consider many factors that affect customer attitudes in CRM activities before launching CRM activities (Luo and Lv, 2019).

However, with the increasing diversification of enterprise CRM activities, this type of business-consumer-based transaction activities that regard CRM as merely a horizontal cooperation model between enterprises and charities or non-profit organizations has certain limitations (Sun and Wang, 2017). In view of this, scholars have further expanded the research scope of enterprise CRM, proposing that CRM covers transaction-based promotional activities, brand authorization activities and donation activities (Andreason, 1996). Based on the perspective of economic value and social value creation, Sun and Wang (2017) divided enterprise CRM into four types, such as transactional, relational, social and public welfare, and proposed corresponding measures and marketing strategies for different types of enterprise CRM activities.

On the one hand, the existing research results mostly focus on the driving factors of enterprise CRM and their influences, such as corporate size, corporate image, involvement, fitness and donation on the organizational side, product price premium at the product level, and sales confidence at the employee level, etc. Research on the relationship between the results of enterprise cause-related marketing (consumers’ perception, attitude or response to CRM) (Anuar and Mohamad, 2011; Jiang and Zheng, 2017; Si, 2017; De Vries and Duque, 2018; Luo and Lv, 2019; Zhang et al., 2020; Mo, 2021); on the other hand, it mainly explores the influence mechanism of consumers’ recognition or attitude toward enterprise CRM on their purchase intention (Sun and Wang, 2016; Duarte and Silva, 2018; Sung et al., 2020; Ye et al., 2021). However, the existing researches on enterprise CRM perception and customer extra-role behavior are relatively scarce. Therefore, this research is to explore the influence mechanism of enterprise CRM perception on customer recommendations, helping people and feedback based on the attribution theory in order to clarify the customer’s behavioral feedback effect on the enterprise’s CRM.

### Customer Extra-Role Behavior

Organizational behavior theory believes that corporate employee behavior includes both intra-role behavior and extra-role behavior. Intra-role behavior is the behavior that employees must take to perform their job duties. If they can’t complete it on time or take relevant behaviors, they will be punished accordingly (Katz, 1964). While the customer extra-role behavior refers to the activities or behaviors taken by employees voluntarily outside the scope of work (Organ and Ryan, 1995). Western research on customer extra-role behavior originates from the employee extra-role behavior theory from the perspective of organizational behavior. Groth (2001) refers to the related research results of organizational behavior theory and proposes that customer behavior can also be divided into two types, such as intra-role behavior and extra-role behavior. Intra-role behavior refers to the behavior shown by customers to complete service production and delivery. Extra-role behavior is the behavior that is not necessary for customers’ spontaneous production or service to be successfully delivered. Customer extra-role behavior is a kind of customer citizenship behavior, which mainly includes three dimensions of recommendation (REC), help (HELP), and feedback (FB) (Groth, 2005; Sha et al., 2013; Elif et al., 2016). Among them, recommendation refers to customers actively recommending related products or services to relatives, friends or other people. Helping means that customers will actively help other customers to search for company product information and explain how to use products or services correctly, and feedback means that customers can actively cooperate with the company’s related research activities and provide the company with product or service optimization suggestions to help the company improve its production process or service delivery process (Groth, 2005).

At present, the research on customer extra-role behavior is still in the exploratory stage. Existing research results mainly explore the influence of organizational behaviors such as organizational justice and social responsibility fulfillment (Xie et al., 2008; Elif et al., 2016) and personal behaviors such as customer loyalty on customer extra-role behaviors (Bettencourt, 1997; Bartikowski and Walsh, 2009). It is not completely customer-oriented, and explains the differentiated influence of enterprise CRM on customer extra-role behavior on the perspective of customer perception. Based on this, this research attempts to explore the different effects of enterprise CRM perceptions on customer recommendations, helping others and feedback in order to better clarify the generation mechanism of customer extra-role behavior.

## Theoretical Basis and Research Hypothesis

### The Influence of Enterprise Cause-Related Marketing on Customer’s Extra-Role Behavior

The enterprise CRM activities can not only provide customers with basic product utility, but also promote consumers to produce better self-perceived emotions (Luo and Lv, 2019). Attribution theory believes that people always like paying attention to the reasons behind events or behaviors, and will perceive, deduce and judge the reasons based on relevant information (Zhang et al., 2020). Individual behavior motivations mainly include internal attribution and external motivations (Sun and Wang, 2016). For example, internal motivation is when an employee helps other employees solve problems in a work environment, they may really want to help others. While the external motivation is that they may be forced to help other employees due to the pressure of the company’s internal regulations.

Cause-related marketing is a complex behavior for enterprises to fulfill their social responsibilities, and customers will also make complex attributions to the motivation or purpose of CRM (Marketing, 2014). In enterprise CRM activities, the internal attribution is that customers believe that the enterprise CRM activities stem from satisfying their own interests, improving corporate reputation, product attractiveness and sales; while the external motivation refers to that the fundamental purpose of enterprises to carry out CRM activities is to fulfill social responsibilities, help solve social problems, and then enhance social value (Myers and Kwon, 2013). Customers prefer companies that have real philanthropic motives. Customers’ judgment on corporate philanthropic motives will affect their enthusiasm for responding to corporate beneficiary marketing activities (Drumwright, 1996). If a company promotes its corporate image and increases sales profits by carrying out CRM activities, but fails to fully fulfill its social responsibilities and pay attention to public welfare events, customers will judge the company’s CRM activities as hypocritical acts of using public welfare events for personal gain (Fan and Ian, 2017). Therefore, customers can judge the real motivation behind the enterprise CRM activities based on their own perceptions of enterprise CRM donation to the society and their attention to and involvement in public welfare events (Zhang et al., 2020). Different perceptions and judgments of customers on the marketing motives of the enterprise will affect their attitudes, which in turn affects the good image of the enterprise in the minds of customers (Luo and Lv, 2019).

In enterprise CRM activities, when customers perceive that the company has carried out CRM activities for the purpose of maximizing its own profits by increasing product sales, that is, the enterprise CRM activities are attributed to self-service motives such as the increase in corporate profits. As a result, customers tend to strengthen their suspicion or negative attitudes toward the company (Rifon et al., 2004), and reduce their trust and loyalty to the company’s brand and its products (Pharr and Lough, 2012). Customers will not actively recommend corporate products to their relatives and friends, help other customers understand the company and its product information and usage rules, and will not provide corporate marketing departments or personnel with suggestions for product or service improvement. It is difficult for companies to get better results of product using feedback ultimately which led to the failure of enterprise CRM (Elif et al., 2016). In summary, this research proposes hypotheses H1 and sub-hypotheses H1a, H1b, and H1c.

H1: Enterprise egoistic cause-related marketing (ECRM) has a significant negative impact on customer extra-role behavior.

H1a: Enterprise ECRM has a significant negative impact on customer recommendation.

H1b: Enterprise ECRM has a significant negative impact on customer help.

H1c: Enterprise ECRM has a significant negative impact on customer feedback.

However, when customers perceive that the company conducts CRM for altruistic motives, on the one hand, customers will have a significant positive impact on customer brand selection and product evaluation (Moosmayer and Fuljahn, 2013); on the other hand, customers will be more supportive of cause-related events. This will increase the sales volume and profit of the company’s products (Youn and Kim, 2018). For example, factors such as the high fit degree between cause-related events and companies and the large amount of corporate donations will prompt customers to attribute enterprise CRM to altruistic motives, which positively affects customers’ willingness and behavior to participate in CRM activities. In addition, enterprise altruistic cause-related marketing (ACRM) can stimulate customers to actively play the role of corporate “part-time employees” (Groth, 2005), recommend corporate products or services to other customers, and enthusiastically help other customers understand corporate culture, products and services, and give positive feedback to the product and service research questions raised by the sales staff of the enterprise (Elif et al., 2016). In summary, enterprise ACRM is more likely to induce customers to feel the social value brought by the company’s cause-related marketing activities, stimulate customers’ sense of moral identity and generate extra-role behaviors. In view of this, this research proposes hypotheses H2 and sub-hypotheses H2a, H2b, and H2c.

H2: Enterprise ACRM has a significant positive impact on customer extra-role behavior.

H2a: Enterprise ACRM has a significant positive impact on customer recommendation.

H2b: Enterprise ACRM has a significant positive impact on customer help.

H2c: Enterprise ACRM has a significant positive impact on customer feedback.

### The Moderating Effect of Customer Regulatory Focus

To a certain extent, the generation of customer extra-role behavior depends on the customer’s attitude, and the customer’s attitude is formed by the customer’s own processing. Customers are the participants and evaluators of enterprise cause-related marketing activities, and their individual characteristics can influence customers’ attitudes toward cause-related marketing activities (Luo and Lv, 2019). Promoting focus customers pay more attention to the positive benefits generated by the company’s product information, they pay more attention to their own progress and growth, and they are full of expectations for the future results of the simulation. While the prevention focus customers pay more attention to negative conditions and avoid damages. They form a robust functional understanding and value evaluation of the company and its products through effective experience analogy, and therefore they tend to adopt evasive behavior strategies (Huang et al., 2020).

In addition, individuals with different regulatory focus have different behavioral strategies and emotional experiences in the process of making behavioral decisions or pursuing goals (Idson et al., 2004). Promotion focus individuals are more able to identify with the enterprise behaviors and tend to maintain a good relationship with the enterprise. This identification is conducive to strengthening the individual’s self-esteem and self-enhancement consciousness. The more an individual recognizes the enterprise, the easier it is to encourage the individual to provide valuable feedback information to the enterprise and strengthen the cooperation between the two parties (Sha et al., 2013). However, prevention focus individuals pay more attention to whether there are negative results behind corporate behaviors, and tend to adopt vigilant motivation strategies. Therefore, tasks or results with a high probability of success can effectively reduce the level of vigilance motivation for prevention focus individuals and show lower performance (Yao and Le, 2009). It can be seen that in view of the different concerns of individuals with different regulatory focus, the individual emotion, cognitive results and behavioral responses triggered by the marketing environment will also show different characteristics (Lee et al., 2010; Li et al., 2014).

In the enterprise cause-related marketing activities, on the one hand, when the enterprise conducts cause-related marketing activities out of self-interested motives, the characteristics of customers promotion focus is more obvious, the more they pursue the positive results of enterprise cause-related marketing, and they are more willing to believe in the purpose of enterprise cause-related marketing activities is to fulfill social responsibilities or create social value. Promotion focus customers believe that companies can indeed donate a certain amount of product sales revenue to charities through cause-related marketing activities, so they tend to support and actively participate in enterprise cause-related marketing activities (Wang et al., 2020), and recommend corporate products or services to other personnel, to help other customers familiarize themselves with the company’s products, understand the product or service usage specifications, and voluntarily provide feedback to the company on product or service improvement issues. It can be seen that customer promotion focus can weaken the negative effect of enterprise ECRM on customer extra-role behavior to a certain extent, that is, customer promotion focus has a significant positive moderating effect in the negative influence of enterprise ECRM on customer extra-role behavior. On the other hand, when an enterprise conducts cause-related marketing out of altruistic motives, the promotion focus customers are more convinced that the purpose of the enterprise cause-related marketing activities is to benefit the society and fulfill corporate social responsibilities. Therefore, the promotion focus customers are more able to identify with the enterprise behaviors (Anna and Joerg, 2018; Coleman et al., 2019), consciously act as a “part-time employee,” actively recommend company products and services to relatives, friends or colleagues around them, help others solve problems in product using, and actively cooperate with the interview work of enterprise sales staff about product or service usage opinions. In summary, customer promotion focus strengthens the positive impact of enterprise ACRM on customer extra-role behavior, that is, customer promotion focus also plays a significant positive moderating effect role in the positive impact of enterprise ACRM on customer extra-role behavior. Based on this, this research proposes hypothesis H3 and its sub-hypotheses H3a and H3b.

H3: Customer promotion focus has a significant positive moderating effect in the influence of enterprise cause-related marketing on customer extra-role behavior.

H3a: Customer promotion focus has a significant positive moderating effect in the negative influence of enterprise ECRM on customer extra-role behavior.

H3b: Customer promotion focus has a significant positive moderating effect in the positive influence of enterprise ACRM on customer extra-role behavior.

However, prevention focus customers pay more attention to the negative results caused by corporate behavior, and are more sensitive to negative information, so they tend to adopt conservative avoidance strategies or behaviors (Sha et al., 2013). On the one hand, when an enterprise conducts cause-related marketing activities for the purpose of increasing product sales and obtaining more economic profits, prevention focus customers will immediately search for relevant information to infer the enterprise’s self-interested motives (Cowan and Yazdanparast, 2019) and believe that the enterprise cause-related marketing is solely to improve its own economic efficiency, not to fulfill its social responsibilities. Based on this, the prevention focus customers will often have a sense of disgust and distrust toward the hypocritical behavior of enterprises using public welfare for their own benefit. Prevention focus customers tend to choose to adhere to the obligations and responsibilities of social citizens, and will not be involved in enterprise cause-related marketing events at all, and will not actively promote or recommend corporate products to relatives and friends, help other customers familiarize themselves with product usage rules, or cooperate with the feedback on enterprise-related products. It can be seen that customer prevention focus can strengthen the negative effect of enterprise ECRM on customer extra-role behavior to a certain extent, that is, customer prevention focus has a significant negative moderating effect in the negative impact of enterprise ECRM on customer extra-role behavior. On the other hand, when the enterprise is really carrying out cause-related marketing activities for public welfare events, customers with obvious precautionary characteristics will still collect information to assess the true motivation of the enterprise cause-related marketing, so as to avoid potential risks or other negative effects of the enterprise cause-related marketing (Sunaga et al., 2020). In addition, even though prevention focus customers are perceiving the altruistic motives of the enterprise cause-related marketing, they still find it difficult to voluntarily perform the role of “part-time employees,” and they have a certain skepticism toward the enterprise cause-related marketing. As a result, they will not sincerely recommend the company products or services to other groups, help others solve product usage problems, or actively feedback product usage opinions to the sales staff. In summary, customer prevention focus weakens the positive impact of enterprise ACRM on customer extra-role behavior, that is, customer prevention focus plays a significant negative moderating role in the positive impact of enterprise ACRM on customer extra-role behavior. Based on this, this research proposes hypothesis H4 and its sub-hypotheses H4a and H4b.

H4: Customer prevention focus has a significant negative moderating effect on the impact of enterprise cause-related marketing on customer extra-role behavior.

H4a: Customer prevention focus has a significant negative moderating effect in the negative influence of enterprise ECRM on customer extra-role behavior.

H4b: Customer prevention focus has a significant negative moderating effect in the positive impact of enterprise ACRM on customer extra-role behavior.

**FIGURE 1:**
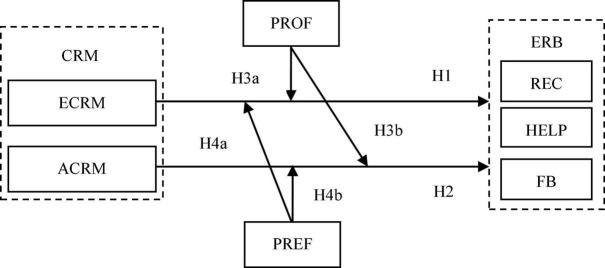
Theoretical model.

The theoretical model of this research is shown in Figure 1.

## Research Design

This experiment adopts 2 (enterprise cause-related marketing: egoistic and altruistic) × 2 (regulatory focus: promotion & prevention) experimental design. The experimental process includes two stages: pre-test and post-test. The pre-test stage is mainly based on the traits regulatory questionnaire, and the experimental subjects are divided into the promotion focus group and the prevention focus group. In the post-test phase, the subjects were asked to read the enterprise’s news events related to different cause-related marketing motives, and carefully fill in the questionnaires about the enterprise cause-related marketing motivation perception and customer extra-role behavior.

In this experiment, the cause-related marketing behavior of a certain beverage enterprise was selected as the experimental target, and the experimental object was a college student who majored in “Marketing” in a certain university, in order to eliminate the interference of knowledge differences on the results of this experiment. The specific steps of this experiment are as follows:

Step 1: grouping. The subjects were divided into two groups (A promotion focus group and B prevention focus group) by asking them to fill out the trait regulatory questionnaire.

Step 2: Introduction to the basic information of the company. After the grouping is over, professionals will broadcast the corporate promotional articles to learn about the company’s products, main business and corporate culture.

Step 3: Separation of the group. In order to ensure that the experimental subjects can complete the experiment independently and make relatively rigorous decisions, the two groups were arranged to participate in the experimental activities in different classrooms.

Step 4: Situational simulation. This scenario simulation consists of two phases. In the first phase, the subjects of group A and group B were asked to watch a news event: 3 years ago, the beverage company was in an advertising campaign in order to quickly open up sales and occupy the market. It promised to use 5% of its annual profit to support Chinese public welfare undertakings, but after media reports, the beverage company did not fulfill this promise. After a break, in the second stage, the subjects of group A and group B were asked to watch another news event: the beverage company changed a new marketing director, recently launched a new marketing plan, and decided to donate a certain percentage of the sales of each bottle of beverage sold to the impoverished mountainous areas of China to support the education of left-behind children in impoverished areas. At the end of each stage, the subjects were asked to fill in the corresponding questionnaire carefully.

Among them, this experiment draws on the questionnaires of Yoon et al. (2006) and Zhang et al. (2020) on enterprise cause-related marketing behaviors, and designed four measurement items, such as “the company is to make better use of social welfare undertakings to achieve its own sales and profit growth, the company sincerely wants to support social welfare undertakings” etc.; drawing lessons from Groth (2005) and Sha et al. (2013) on customer extra-role behavior measurement questionnaires, 11 items were designed to measure customer extra-role behavior from the three levels of recommending, helping other customers and providing feedback, such as “you will recommend this company and its related products to friends and relatives, you will actively help other customers to use the company’s products or enjoy services, and you will provide the company with suggestions for improving products or services” etc.; drawing on the content of the regulatory focus questionnaire proposed by Higgins et al. (2001), six items to measure customer promotion focus and five items to measure customer prevention focus were designed respectively, such as “you feel that you have achieved in your life, you often annoy your parents when you grow up” and so on. In addition, considering that individual characteristics may have a certain influence on their behavior, this experiment uses the gender and average monthly consumption level of the experimental subjects as the control variables.

## Empirical Results and Analysis

### Sample Statistics

In this experiment, a total of 124 college students were recruited as experimental subjects with an average age of 21 years. According to the trait regulatory questionnaire filled out by the subjects in the pre-test phase, this experiment found that 76 subjects were clHELPified into the promotion focus group (group A), and 48 subjects belonged to the prevention focus group (group B). In the promotion focus group, men accounted for 40.8% (31 people) and women accounted for 59.2% (45 people); in the prevention focus group, men accounted for 39.6% (19 people) and women accounted for 60.4% (29 people).

### Reliability and Validity Test

This research uses SPSS17.0 to test the reliability and validity of the questionnaire data of each measurement scale. The test results are shown in Table 1. The minimum Cronbach’s α value of each measurement scale is 0.816, which is greater than 0.7, indicating that each scale has good reliability; the minimum KMO value is 0.724, which is also greater than 0.7, indicating that each scale has good structural validity and each scale has good reliability. The significance of the Bartlett sphere chi-square test of the variables is *p* = 0.000 < 0.001, indicating that the original variables in each model have a high degree of interpretation and good validity, so subsequent correlation analysis can be carried out.

**TABLE 1:** Reliability and validity test results.

Variables	Dimensions	Cronbach’s α	KMO	Bartlett sphere chi-square test
Cause-related marketing	Egoistic	0.924	0.836	249.87(*p* = 0.000)
	Altruistic	0.882	0.809	109.13(*p* = 0.000)
Extra-role behavior	Recommendation	0.816	0.774	65.17(*p* = 0.000)
	Help	0.957	0.724	381.61(*p* = 0.000)
	Feedback	0.866	0.791	97.29(*p* = 0.000)
Regulatory focus	Promotion focus	0.842	0.875	187.72(*p* = 0.000)
	Prevention focus	0.932	0.830	305.42(*p* = 0.000)

### Main Effect Test

In order to verify that enterprise cause-related marketing has a significant impact on customer extra-role behavior, this research conducted a one-way variance analysis. As shown in Table 2, for the promotion focus group of group A, the smallest explainable variation of egoistic marketing is 5.61, and the smallest explainable variation of altruistic marketing is 1.59, and *p* < 0.05, indicating that egoistic marketing and altruistic marketing has a significant impact on customer extra-role behavior (recommendation, help, and feedback), and its effect on customer extra-role behavior is not all zero. Through further regression analysis, this research finds that egoistic marketing has a significant negative impact on customer extra-role behavior (β_recommendation_ = –0.323, β_help_ = –0.290, β_feedback_ = –0.246, *p* < 0.05), while altruistic marketing exerts a significant positive effect on customer extra-role behavior (β_recommendation_ = 0.335, β_help_ = 0.310, β_feedback_ = 0.316, *p* < 0.05). In the prevention focus group of group B, the least explainable variation of egoistic marketing is 3.16, and *p* < 0.05; the least explainable variation of altruistic marketing is 5.76, and *p* < 0.05, indicating egoistic marketing and altruistic marketing have significant impacts on customer extra-role behaviors (recommendation, help and feedback), and their impacts on customer extra-role behaviors are not all zero. After further regression analysis, it is found that egoistic marketing also has a significant negative impact on customer extra-role behavior (β_recommendation_ = –0.533, β_help_ = –0.492, β_feedback_ = –0.471, *p* < 0.05), while altruistic marketing also plays a significant positive role in customer extra-role behavior (β_recommendation_ = 0.670, β_help_ = 0.520, β_feedback_ = 0.440, *p* < 0.05). To sum up, H1 and its sub-hypotheses H1a, H1b, and H1c, and H2 and its sub-hypotheses H2a, H2b, and H2c all pass the test. In addition, compared with egoistic cause-related marketing (*M*_*A*__recommendation_ = 2.05, *M*_*A*__*help*_ = 2.05, *M*_*A*__feedback_ = 2.00; *M*_*B*__recommendation_ = 1.79, *M*_*B*__*help*_ = 1.79, *M*_*B*__feedback_ = 1.82, *p* < 0.05). Altruistic cause-related marketing can more motivate customers to have extra-role behaviors (*M*_*A*__recommendation_ = 4.43, *M*_*A*__*help*_ = 4.45, *M*_*A*__feedback_ = 4.32; *M*_*B*__recommendation_ = 4.25, *M*_*B*__*help*_ = 4.26, *M*_*B*__feedback_ = 4.22, *p* < 0.05).

**TABLE 2:** Results of one-way variance analysis.

Variables	A one-way variance analysis				Regression coefficients	B one-way variance analysis				Regression coefficients
	Between groups	Within groups	*M*	*SD*		Between groups	Within groups	*M*	*SD*	
ECRM-REC	8.14	34.40	2.05	0.75	−0.323**	3.58	7.59	1.79	0.49	−0.533***
ACRM-REC	2.11	15.10	4.43	0.48	0.335**	8.08	6.92	4.25	0.56	0.670***
ECRM-HELP	6.76	30.58	2.05	0.71	−0.290*	5.10	12.60	1.79	0.61	−0.492**
ACRM-HELP	2.31	18.93	4.45	0.53	0.310**	5.76	10.52	4.26	0.59	0.520***
ECRM-FB	5.61	29.83	2.00	0.69	−0.246*	3.16	9.58	1.82	0.52	−0.471**
ACRM-FB	1.59	12.76	4.32	0.44	0.316**	6.19	10.97	4.22	0.60	0.440**

***p < 0.001, **p < 0.01, *p < 0.05.

### Moderating Effect Test

As shown in Table 3, customer promotion focus plays a significant positive moderating role in the negative impact of egoistic marketing on customer extra-role behavior (β_recommendation_ = 0.374, β_help_ = 0.411, β_feedback_ = 0.385, *p* < 0.01), that is, customer promotion focus weakens the negative effect of egoistic marketing on customer extra-role behavior. Therefore, hypothesis H3a passes the test. Customer promotion focus also has a significant positive moderating effect in the positive influence of altruistic marketing on customer extra-role behavior (β_recommendation_ = 0.426, β_help_ = 0.419, β_feedback_ = 0.427, *p* < 0.001), that is, customer promotion focus strengthens the positive effect of altruistic marketing on customer extra-role behavior, and H3b passes the test.

**TABLE 3:** Test results of the moderating effect of promotion focus.

Variables/model	Recommendation		Help		Feedback	
	Model 1	Model 2	Model 3	Model 4	Model 5	Model 6
**Control variables**						
Gender	0.069	0.057	0.109	0.085	0.099	–0.148
Consumption	0.072	–0.101	0.011	–0.127	–0.006	–0.052
**The moderating effect of promotion focus**						
Egoistic marketing	−0.477***		−0.459***		−0.405**	
Altruistic marketing		0.149		0.127		0.129
ECRM*PROF	0.374**		0.411**		0.385**	
ACRM*PROF		0.426***		0.419***		0.427***
Adjusted *R*^2^	0.190	0.232	0.196	0.217	0.150	0.246
*F*	5.405**	6.649***	5.578**	6.205***	4.304**	7.112***

***p < 0.001, **p < 0.01, *p < 0.05.

As shown in Table 4, customer prevention focus plays a significant negative moderating role in the negative impact of egoistic marketing on customer extra-role behavior (β_recommendation_ = –0.362, β_help_ = –0.483, β_feedback_ = –0.504, *p* < 0.05), that is, customer prevention focus strengthens the negative effect of egoistic marketing on customer extra-role behaviors. Therefore, hypothesis H4a can be verified. Customer prevention focus has a significant positive moderating effect in the positive influence of altruistic marketing on customer extra-role behaviors (β_recommendation_ = 0.388, β_help_ = 0.377, β_feedback_ = 0.449, *p* < 0.05), that is, customer prevention focus strengthens the positive effect of altruistic marketing on customer extra-role behavior. Hypothesis H4b fails the test. In summary, hypothesis H3 is verified, while hypothesis H4 only partially passes the test.

**TABLE 4:** Test results of the moderating effect of prevention focus.

Variables/model	Recommendation		Help		Feedback	
	Model 1	Model 2	Model 3	Model 4	Model 5	Model 6
**Control variables**						
Gender	0.021	–0.025	0.108	–0.053	0.091	–0.066
Consumption	–0.050	–0.028	0.113	–0.095	0.108	0.134
**The moderating effect of prevention focus**						
Egoistic marketing	–0.268		–0.139		–0.102	
Altruistic marketing		0.387*		0.245		0.112
ECRM*PREF	−0.362*		−0.483**		−0.504**	
ACRM*PREF		0.388*		0.377*		0.449*
调整的*R*^2^	0.282	0.477	0.302	0.292	0.290	0.256
*F*值	5.608**	11.703***	6.082**	5.837**	5.791**	5.039**

***p < 0.001, **p < 0.01, *p < 0.05.

## Research Conclusion and Discussion

### Conclusion

Based on attribution theory and regulatory focus theory, this research explores the influence of enterprise cause-related marketing on customer extra-role behavior, and examines the moderating effect of customer promotion focus and customer prevention focus. The research results show that enterprise ECRM negatively affects customer extra-role behavior, while enterprise ACRM positively affects customer extra-role behavior, that is, enterprise cause-related marketing has a differentiated effect on customer extra-role behavior. In addition, customer promotion focus weakens the negative effect of enterprise ECRM on customer extra-role behavior, and strengthens the positive effect of enterprise ACRM on customer extra-role behavior. However, the customer prevention focus strengthens the negative effect of enterprise ECRM on customer extra-role behavior and the positive effect of enterprise ACRM on customer extra-role behavior.

### Discussion

This research reveals the influence mechanism of enterprise cause-related marketing on customer extra-role behaviors, which is conducive to the advancement of the understanding of the relationship between enterprise cause-related marketing and customer extra-role behaviors. Although existing studies have explored the relationship between enterprise cause-related marketing and customer attitudes and behaviors, they are mostly based on the internal perspective of customers to discuss the generation mechanism of intra-role behaviors such as customer purchase intentions (Luo and Lv, 2019; Zhang et al., 2020; Mo, 2021). Cause-related marketing is a complex behavior for enterprises to fulfill their social responsibilities, and customers often make complex attributions to the motivation or purpose of cause-related marketing (Marketing, 2014). Based on the attribution theory, this research finds that enterprise ECRM is not conducive to stimulating customers to have extra-role behaviors, while enterprise ACRM is conducive to stimulating customers to produce extra-role behaviors and play the role of corporate “part-time employees” better. This provides new perspectives and insights for understanding how enterprise cause-related marketing inspires customers to have extra-role behaviors.

This research clarifies the role of customer regulatory focus on the relationship between enterprise cause-related marketing and customer extra-role behaviors, and helps academia understand the effective mechanism of successful enterprise cause-related marketing based on a contingency perspective. The specific results show that customer promotion focus can weaken the negative effect of enterprise ECRM on customer extra-role behavior, and strengthen the positive effect of enterprise ACRM on customer extra-role behavior. However, the customer prevention focus can strengthen the negative effect of enterprise ECRM on customer extra-role behavior and the positive effect of enterprise ACRM on customer extra-role behavior. With the changes in the external market environment, the competition among enterprises has become increasingly fierce, and customers have put forward higher requirements for enterprises to fulfill their social responsibilities. Customers are the participants and evaluators of enterprise cause-related marketing activities, and their individual characteristics can affect customers’ attitudes or behaviors toward cause-related marketing activities (Luo and Lv, 2019). Based on the perspective of individual customer characteristics, this research reveals the boundary conditions of enterprise cause-related marketing on customer extra-role behavior, and promotes the dialogue and integration of the two research fields of enterprise cause-related marketing and customer behavior theory.

### Marketing Strategy Enlightenment

Enterprise cause-related marketing is not only a commercial activity that can generate economic profits, but also a social marketing strategy that can bring social value to the enterprise. The results of this research provide certain management enlightenment for enterprise cause-related marketing practice. (1) Pay attention to the development and implementation of enterprise ACRM. Because enterprise ACRM is conducive to customers’ extra-role behaviors, enterprises should pay more attention to public welfare undertakings when carrying out cause-related marketing activities, emphasizing that the fundamental purpose of enterprise cause-related marketing is to fulfill social responsibilities by serving as a charity or other non-profit organization donations to create more social value. (2) Pay attention to the cultivation and construction of customers’ psychological security. Since the prevention focus customers has a “conservative mentality” and tends to adopt risk-avoidance strategies, companies should communicate with customers more. Companies can share the relevant content of enterprise cause-related marketing by building an information platform, and understand the relationship between the company and public welfare issues, so as to enhance customers’ psychological security and perception of trust in the company, and improve their involvement of enterprise cause-related marketing events. (3) Pay attention to the guidance of customer extra-role behavior. In view of the fact that customer extra-role behavior is an important measurement standard for the success of a company’s cause-related marketing, companies should fully consider the factors that affect customers’ extra-role behavior before launching cause-related marketing activities, and establish a benefit link mechanism between cause-related marketing events and customers, so as to stimulate customers to have extra-role behaviors and take the initiative to assume the responsibilities of “part-time employees.”

### Limitations and Future Research Directions

Although this research has enriched the results of customer behavior theory and regulatory focus theory to a certain extent, and provides useful reference for enterprises to successfully carry out cause-related marketing activities, it still has limitations. On the one hand, the experimental objects in this research are college undergraduates. Although it is convenient to control the influence of some variables on the results of experiments and empirical analysis, it also limits the broadness of the research objects. Follow-up research can broaden the scope of research objects, and carry out research on the relationship between enterprise cause-related marketing and customer extra-role behaviors of diversified social groups, so as to enhance the universality of research conclusions. On the other hand, based on the customer’s perception of enterprise cause-related marketing motivation, this research divides enterprise cause-related marketing into ECRM and ACRM, ignoring that other dimensions or factors of cause-related marketing have an impact on the customer extra-role. The follow-up research can further explore the differentiating effects of different types of cause-related marketing on customer extra-role behavior based on the perspective of value creation or the characteristics of enterprise cause-related marketing. In addition, follow-up research can also explore the mechanism of enterprise cause-related marketing on customer behavior. By comparing the effects of enterprise cause-related marketing on customer intra-role behavior and extra-role behavior, clarify the evolution mechanism of the company cause-related marketing activities and the complex behaviors of customers. In the end, it provides useful theoretical reference value for enterprises to successfully carry out cause-related marketing activities and fulfill social responsibilities.

## Data Availability Statement

The original contributions presented in the study are included in the article/supplementary material, further inquiries can be directed to the corresponding author.

## Ethics Statement

Ethical review and approval was not required for the study on human participants in accordance with the local legislation and institutional requirements. Written informed consent for participation was not required for this study in accordance with the national legislation and the institutional requirements.

## Author Contributions

ZH and HW contributed to the content writing and data processing of this article. Both authors contributed to the article and approved the submitted version.

## Conflict of Interest

The authors declare that the research was conducted in the absence of any commercial or financial relationships that could be construed as a potential conflict of interest.

## Publisher’s Note

All claims expressed in this article are solely those of the authors and do not necessarily represent those of their affiliated organizations, or those of the publisher, the editors and the reviewers. Any product that may be evaluated in this article, or claim that may be made by its manufacturer, is not guaranteed or endorsed by the publisher.
